# Comparative Study of Vaginal Hysterectomy and Total Abdominal Hysterectomy in Non-descent Uterus in a Rural Tertiary Care Center

**DOI:** 10.7759/cureus.36017

**Published:** 2023-03-11

**Authors:** Divya N Alamelu, Bharathi K.R, Sridhar D, Vijayalakshmi S

**Affiliations:** 1 Obstetrics and Gynaecology, Mahatma Gandhi Medical College and Research Institute, Sri Balaji Vidyapeeth (Deemed to be University), Puducherry, IND; 2 Obstetrics and Gynaecology, Adichunchanagiri Institute of Medical Sciences, B.G.Nagara, IND; 3 Community and Family Medicine, Mahatma Gandhi Medical College and Research Institute, Sri Balaji Vidyapeeth (Deemed to be University), Puducherry, IND

**Keywords:** benign tumors, complications, rural center, non-descent uterus, vaginal hysterectomy, total abdominal hysterectomy

## Abstract

Aim

To study the role of vaginal hysterectomy in non-descent uterus and to compare it with abdominal hysterectomy with respect to operative time, intraoperative blood loss and complications, ambulation, and postoperative complications.

Materials and methods

A prospective non-randomized study was carried out on 200 cases at a rural tertiary care center in B.G. Nagara, Karnataka, India for a period of 18 months after obtaining institutional ethical committee approval. One hundred patients underwent a vaginal hysterectomy, and there other 100 underwent an abdominal hysterectomy for similar indications.

Results

Mean age, parity, mode of delivery, BMI, uterine size, and anesthesia were similar between the groups. The most common indication was fibroid uterus (50%). It was found that the vaginal hysterectomy group was associated with significantly reduced mean operative duration and a decline in postoperative Hemoglobin when compared to the abdominal hysterectomy group. Patients who underwent vaginal hysterectomy had less postoperative pain and were ambulated earlier and discharged earlier. Also, postoperative complications were more common in those who underwent abdominal hysterectomy.

Conclusion

Vaginal hysterectomy is a safe and the least invasive route and is associated with lesser complications and should be chosen as the preferred method of hysterectomy, whenever feasible.

## Introduction

This article was presented as a paper in the "Gestosis-2022", 7th Annual conference of Gestosis India Association, Pondicherry held on 19-20, November 2022. 

Hysterectomy is the most frequently performed major gynecological operative procedure throughout the world [1]. Hysterectomy is performed vaginally, abdominally, or with laparoscopic or robotic assistance.

Abdominal hysterectomy is the most widely performed technique worldwide and accounts for 66% of cases, while vaginal hysterectomy and laparoscopic hysterectomy account for 22% and 12% of cases respectively [1]. With the advent of laparoscopic hysterectomy, minimally invasive surgeries have come to the limelight. Vaginal hysterectomy is associated with lesser morbidity, early ambulation, reduced hospital stay, and reduced cost when compared to abdominal hysterectomy and laparoscopic hysterectomy. Despite the advantages of vaginal hysterectomy, abdominal hysterectomy is still the most commonly performed technique worldwide, being associated with increased complications, higher costs, and longer hospital stay. Laparoscopic hysterectomy is constantly gaining ground but is associated with increased costs, longer duration of surgery, and involves trained and skilled personnel. Therefore, there is a need for expanding the indication of vaginal hysterectomy rather than limiting it to the conventional indication of uterovaginal prolapse [2,3].

Large uterine size, which is considered by many gynecologists as a contraindication for vaginal hysterectomy, can be facilitated by techniques like bisection, myomectomy, intramyometrial coring, and morcellation. The other common limitations considered for vaginal hysterectomy are nulliparity and previous cesarean section. These can however be overcome by surgical expertise and cautious operative techniques. Therefore, there is a need for a renaissance of vaginal hysterectomy, a safe, cost-effective minimally invasive surgery that is associated with fewer complications and should be the preferred surgical approach whenever feasible.

The aim of our study was to ascertain the role and feasibility of vaginal hysterectomy in non-descent uterus and to compare it with abdominal hysterectomy with respect to operative duration, intraoperative blood loss, intra-operative complications, ambulation, postoperative complications, and hospital stay.

## Materials and methods

All patients meeting the inclusion and exclusion criteria requiring hysterectomy in a rural tertiary care center in B.G. Nagara, Karnataka, India, were enrolled in the study between August 2014 and January 2016. It was a non-randomized prospective study conducted after obtaining the institutional ethical committee approval from Adhichunchanagiri Insitute of Medical Sciences, B.G. Nagara with approval number-01_M009_52974. The inclusion criteria were all non-descent uteri undergoing hysterectomy with uterine size less than 16 weeks gravid uterus size and good uterine mobility. Nulliparous women and women with previous cesarean sections were also included in the study. Uterovaginal prolapse, gynecological malignant disease, adnexal pathology >5 cm, acute pelvic inflammatory disease, and severe endometriosis were excluded from the study.

In our tertiary care center, there were two units of surgeons, of which one unit performed hysterectomy only by the vaginal route, and the other unit only by the abdominal route. Each unit was allocated with different surgeons in all cadres, ranging from senior to junior consultant. However, both teams were of approximately the same level of skills, when compared to one another. Hence, both groups were comparable with respect to all the study parameters. Patients were recruited in the study by purposive sampling method and 200 patients requiring hysterectomy for benign gynecological disorders were selected for the study. One hundred patients underwent vaginal hysterectomy operated by one surgical unit, and one hundred patients underwent abdominal hysterectomy operated by the other surgical unit. After obtaining written informed consent, cases were subjected to proper pre-operative evaluation with clinical history and examination, and baseline investigations including blood grouping and Rh typing, complete blood count, blood sugar, renal function test, urinalysis, HIV, HbsAg screening, ECG, Chest X-ray, USG abdomen and pelvis, and Pap smear. Endometrial biopsy and cervical punch biopsy were done if indicated.

The type of anesthesia was decided by the anesthetist. Routine surgical prophylaxis antibiotic was administered irrespective of the route of the surgery. The operating time for vaginal hysterectomy was calculated from the time of incision at the cervicovaginal junction to the closure of the vaginal vault. The operating time for a total abdominal hysterectomy was calculated from the time of the skin incision to the closure of the skin incision. The decision to remove the ovaries was made by the surgeon based on the age and the condition of the ovaries. Techniques to reduce the uterine size like bisection, morcellation, and myomectomy were performed during vaginal hysterectomy, whenever it was required to facilitate easy removal of the enlarged uterus. Small-sized uteri were removed entirely. Intraoperative blood loss was estimated by measuring pre-operative and second postoperative day Hemoglobin and Hematocrit values. Any intraoperative complications like injury to the bladder, ureter, and bowel, and hemorrhage were noted. Postoperative complications like secondary hemorrhage, need for re-laparotomy due to bleeding, any form of postoperative infection like wound infection, UTI, respiratory tract infection, and thromboembolism were noted. Time taken for ambulation was also noted. Postoperative pain scoring was done on the third postoperative day using the Numeric pain rating scale. The duration of hospital stay was also noted. Patients were followed up postoperatively after four weeks and the presence of fever, bleeding, discharge, and evidence of vault infection was noted.

The entire patient data was compiled and analyzed using SPSS software, version 17, and a P value of < 0.001 was taken as significant.

## Results

The mean age of the patients who underwent vaginal hysterectomy was 42.32 years, and abdominal hysterectomy was 40.76 years. The mean BMI in the vaginal hysterectomy group and abdominal hysterectomy group was 23.95 kg/m² and 23.82 kg/m², respectively. Table 1 shows the parity distribution in the vaginal hysterectomy and abdominal hysterectomy groups. Two nulliparous women underwent vaginal hysterectomy and six nulliparous women underwent abdominal hysterectomy. Comparison of the groups for parity with the chi-square test revealed no significant difference with a p-value of 0.738.

**Table 1:** Parity of patients who underwent vaginal and total abdominal hysterectomy

Parity	Vaginal Hysterectomy, n (%)	Total abdominal Hysterectomy, n (%)	Chi-square	p-value
Nullipara	2 (2%)	6 (6%)	2.753	0.738
1	7 (7%)	8 (8%)		
2	56 (56%)	53 (53%)		
3	25 (25%)	24 (24%)		
4	9 (9%)	7 (7%)		
5	1 (1%)	2 (2%)		
Total	100	100		

In the vaginal hysterectomy group, 95 patients (96.9%) had a vaginal delivery and 3 patients (3.1%) had undergone a cesarean section. In the abdominal hysterectomy group, 85 patients (90.4%) had delivered by the vaginal route and 9 patients (9.6%) had a cesarean section.

The majority of patients in both groups had a uterine size < 8 weeks size. The distribution of uterine size in both groups is as tabulated in Table 2.

**Table 2:** Uterine size of patients who underwent vaginal and total abdominal hysterectomy

Uterine Size	Vaginal Hysterectomy, n (%)	Total abdominal Hysterectomy, n (%)	Chi-square	p-value
< 8 weeks	58 (58%)	46 (46%)	3.905	0.142
8 – 12 weeks	32 (32%)	36 (36%)		
> 12 weeks	10 (10%)	18 (18%)		
Total	100 (100%)	100 (100%)		

A comparison of the two groups in terms of uterine size showed that no significant difference was present when the chi-square test was applied. The p-value of 0.142 suggests that the uterine size of patients was similar in both groups.

The most common indication for hysterectomy in both groups was fibroid uterus-AUB(L). Salpingo-oophorectomy was performed in 12 cases of vaginal hysterectomy and 61 cases of abdominal hysterectomy. The group-wise distribution of the indication for hysterectomy is illustrated in Table 3.

**Table 3:** Diagnosis of patients who underwent vaginal and total abdominal hysterectomy AUB(L)- Abnormal Uterine Bleeding (Leiomyoma) AUB(A)-Abnormal Uterine Bleeding (Adenomyosis) AUB(E)- Abnormal Uterine Bleeding (Endometrium) DUB-Dysfunctional Uterine Bleeding PID-Pelvic Inflammatory Disease

Diagnosis	Vaginal Hysterectomy, n (%)	Total abdominal Hysterectomy, n (%)	Chi-square	p-value
AUB(L)	50 (50%)	50 (50%)	8.98	0.11
AUB(E)/DUB	26 (26%)	28 (28%)		
AUB(A)	10 (10%)	12 (12%)		
Chronic PID	3 (3%)	8 (8%)		
Endometrial Hyperplasia	9 (9%)	2 (2%)		
Atrophic Endometritis	2 (2%)	0 (0)		
Total	100	100		

The mean weight of the uterus post-surgery in the vaginal hysterectomy group and abdominal hysterectomy group was 120.1 g and 111.5 g, respectively. The weight of the uterus in both groups was comparable (p-value of 0.271 was not statistically significant).

There was no difference in the anesthesia used for the surgery. The majority of patients received spinal anesthesia in both groups. Various techniques were performed for the removal of the uterus by vaginal hysterectomy. They are as follows, as in Table 4.

**Table 4:** Techniques used for patients who underwent vaginal hysterectomy

Technique	Vaginal hysterectomy, n (%)
Bisection	40 (40%)
In Toto	32 (32%)
Morcellation	23 (23%)
Myomectomy	5 (5%)

No major intraoperative complication was noted in both groups. Adhesions were present in two cases of vaginal hysterectomy and eight cases of abdominal hysterectomy. No other complication of bladder, ureter, and bowel injury and hemorrhage was noted.

The mean duration of surgery for vaginal hysterectomy and abdominal hysterectomy was 58.45 min and 87.55 min, respectively. The p-value calculated by the t-statistic test proved to be <0.001, which was statistically significant. The minimum time required to perform a vaginal hysterectomy and abdominal hysterectomy was 35 min and 30 min, respectively. The maximum time taken for a vaginal hysterectomy was 100 min, while that for an abdominal hysterectomy was 160 min.

Intraoperative blood loss was calculated by noting the decline in pre-operative Hemoglobin and the second postoperative day Hemoglobin. The mean pre-operative hemoglobin in the vaginal group was 12 g/dl, and 11.9 g/dl in the abdominal group. The mean pre-operative PCV was 36.09% and 31.31% in the vaginal group and the abdominal group, respectively. The mean postoperative hemoglobin was 10.82 g/dl and 10.51 g/dl in the vaginal group and the abdominal group, respectively. The mean postoperative day-2 PCV in the vaginal group was 32.31%. and 31.54% in the abdominal group. The decline in hemoglobin was found to be 1.21 g/dl in the vaginal group, and 1.45 g/dl in the abdominal group. The p-value was 0.08 and was not statistically significant.

The mean time taken for ambulation in vaginal hysterectomy patients was 21 hrs and in abdominal hysterectomy was 25.7 hrs. The difference was statistically significant according to the t-statistic value test, with a p-value of <0.001.

Pain was measured by the Numeric Pain rating scale on the third postoperative day. The mean score in the vaginal group was 2.79, and 4.11 in the abdominal group. The difference in both groups was statistically significant with a p-value of <0.001 according to the Mann-Whitney test.

The various postoperative complications observed in both groups are tabulated in Table 5. However, there was no statistically significant difference in the occurrence of postoperative complications in both groups.

**Table 5:** Postoperative complications among patients who underwent vaginal and total abdominal hysterectomy UTI-Urinary Tract Infection

Postoperative complication	Vaginal Hysterectomy, n (%)	Total Abdominal Hysterectomy, n (%)	Chi-square	p-value
Pelvic Abscess	1 (1%)	0	8.75	0.12
Pelvic Hematoma	0	1 (1%)		
UTI	2 (2%)	2 (2%)		
Wound Infection	0	5 (5%)		
Wound Gaping	0	3 (3%)		
Abdominal wall Cellulitis	0	1 (1%)		
Total	3 (3%)	12 (12%)		

The mean hospital stay for a vaginal hysterectomy was 6.47 days, and 9.04 days for an abdominal hysterectomy. The difference was statistically significant with a p-value of <0.001.

The patients in both groups were followed up 4 weeks postoperatively. One case of vault infection was noted in both groups. The difference was statistically insignificant with a p-value of 1.

## Discussion

The present study was conducted in a rural tertiary care center in Southern India to evaluate the role of vaginal hysterectomy and compare it with abdominal hysterectomy in a non-descent uterus. In our study, a fibroid uterus was the most common indication for hysterectomy in both groups. The diagnosis for which a hysterectomy was done was comparable to the studies conducted by Chandrika et al and Chakraborty et al [4,5].

Regarding the uterine size, both groups were comparable with respect to their uterine size. The mean weight of the uterus was also compared postoperatively. The difference in the weight between the two groups was not statistically significant, with a p-value of 0.271. The uterine size was comparable to studies by Benassi et al and Taylor et al [6,7].

During a vaginal hysterectomy, various techniques were used to facilitate easy removal of the uterus. The most common technique which was used was bisection (40%). Our study result correlates with Taylor et al study [7].

In the vaginal hysterectomy group, the mean duration of surgery was 58.45 minutes. The operating duration was 87.55 minutes in the abdominal hysterectomy and the difference was statistically significant with a p-value of <0.001. This proved that vaginal hysterectomy was performed faster when compared to abdominal hysterectomy as stated by Shanthini et al and Gayak et al studies [8,9]. This result depends on factors like the size of the uterus, technique used for uterine size reduction, and the experience of the operating surgeon.

There were two cases of adhesions in the vaginal hysterectomy group and eight cases in the abdominal hysterectomy group. However, in our study, we did not have any major intraoperative complications. Studies by Chakraborty et al and Shanthini et al encountered bladder and ureteric injuries in the abdominal hysterectomy group [5,8]. This difference may be attributed to the skill of the operating surgeon and the complexity of the case.

Intraoperative blood loss was calculated by estimating the drop in postoperative Hemoglobin and PCV values in comparison to the pre-operative Hemoglobin and PCV values. The fall in Hemoglobin was estimated to be 1.21 g/dl and 1.45 g/dl in the vaginal and abdominal hysterectomy groups, respectively. The decline in Hemoglobin values was more with the abdominal hysterectomy group, though it was not statistically significant with a p-value of 0.08. Even the study conducted by Hoffman et al concluded that there was greater blood loss in abdominal hysterectomy when compared to vaginal hysterectomy, but it was not statistically significant [10]. However, in the study by Shanthini et al, there was statistically significant blood loss in the abdominal hysterectomy group when compared to vaginal hysterectomy [8]. This difference may be because of encountering any major intraoperative complication. Our study, on the other hand, did not have such major complications. The mean time taken for ambulation in vaginal hysterectomy group was 21 hrs, and in abdominal hysterectomy group, it was 25.7 hrs. The difference in the time to ambulate was statistically significant, with a p-value of <0.001. Our study results are comparable with the study conducted by Chen et al where vaginal hysterectomy patients ambulated after 23.1 hrs when compared to 35.4 hrs in abdominal hysterectomy patients [11]. The study by Chakraborty et al also revealed that 71 % of vaginal hysterectomy patients were mobilized by 24 hrs when compared to 35 % of abdominal hysterectomy patients [5]. Early ambulation in the vaginal hysterectomy group was possible because of reduced postoperative pain. 

In our study, the pain perception on the third postoperative day was significantly less in the vaginal group with a mean Numeric pain score of 2.79 when compared to a 4.11 score in the abdominal group. A similar result was also proved by the study conducted by Goswani et al, where there was less postoperative pain in the vaginal group when compared to the abdominal group [12]. The study by Miskry and Magos also revealed that vaginal hysterectomy patients had reduced analgesic requirements when compared to those who underwent abdominal hysterectomy [13].

The patients who underwent abdominal hysterectomy had more postoperative complications when compared to the vaginal hysterectomy group, though the difference was not statistically significant. It was comparable to the studies by Chandrika et al and Harmanli et al [4,14].

The mean hospital stay was also found to be lower in the vaginal group when compared to the abdominal group. It was 6.47 days in the vaginal hysterectomy group when compared to 9.04 days in the abdominal hysterectomy group, which was statistically significant with a p-value of <0.001. Although patients in the vaginal hysterectomy group were fit for discharge as early as 3-4 days, the rural population prefers to stay in the hospital for a longer duration because of the difficult accessibility to a hospital. They were discharged between 5-7 days. Abdominal hysterectomy patients were usually discharged between 7-9 days after suture removal. These results were comparable to the studies by Shanthini et al and Gayak et al [8,9].

At the four-week follow-up period, one patient in both groups developed a vault infection. Hence, it was not statistically significant. Our study result correlated with the results of the studies by Chandrika et al and Benassi et al [4,6].

The limitation of our study was it was a single-center study. The generalizability of the study results to a larger population should ideally include multiple centers. It was a non-randomized study with purposive sampling, hence there is a chance of selection bias. Randomisation could have prevented the selection bias. Our present study was not done by a single surgeon, hence there was a difference in the technique of each surgery each consultant performed. Surgeries were performed by various surgeons with varying experience, with approximately the same level of skill in both groups. This can produce variability in the operating time with different levels of experience. 

## Conclusions

Vaginal hysterectomy is the least invasive, fast, and least complicated surgery, which can result in early ambulance and the briefest hospital stay at the lowest possible expense when compared to abdominal hysterectomy. The use of the vaginal route of hysterectomy should therefore be extended from the conventional indication for pelvic organ prolapse to a non-descent uterus, even with uterine size up to 16 weeks. With the surgeon’s experience, the operative time, complications and blood loss can be minimized. Therefore, this scar-less, cost-effective, patient-friendly approach should be chosen as the preferred method of hysterectomy, whenever feasible. Worldwide trends are rising toward minimally invasive approaches. Natural orifice techniques of surgery like the vaginal hysterectomy should therefore be the primary choice. And as always, vaginal hysterectomy remains the hallmark of the gynecological surgeon.
